# Lysophosphatidylinositol Promotes Chemotaxis and Cytokine Synthesis in Mast Cells with Differential Participation of GPR55 and CB2 Receptors

**DOI:** 10.3390/ijms24076316

**Published:** 2023-03-28

**Authors:** Lizbeth Magnolia Martínez-Aguilar, Alfredo Ibarra-Sánchez, Daniel José Guerrero-Morán, Marina Macías-Silva, Jesús Omar Muñoz-Bello, Alejandro Padilla, Marcela Lizano, Claudia González-Espinosa

**Affiliations:** 1 Departamento de Farmacobiología Centro de Investigación y de Estudios Avanzados (Cinvestav), Unidad Sede Sur. Calzada de los Tenorios No. 235, Col. Granjas Coapa, Tlalpan, Mexico City 14330, Mexico; magnoliamarths@gmail.com (L.M.M.-A.); aibarra@cinvestav.mx (A.I.-S.); daniel.guerrero@cinvestav.mx (D.J.G.-M.); 2 Departamento de Biología Celular y Desarrollo, Instituto de Fisiología Celular, Universidad Nacional Autónoma de México, Circuito Exterior S/N, Ciudad Universitaria, Mexico City 04510, Mexico; mmacias@unam.mx; 3 Unidad de Investigación Biomédica en Cáncer, Instituto Nacional de Cancerología, Av. San Fernando No 22, Col. Sección XVI, Tlalpan, Mexico City 14080, Mexico; omarmube@gmail.com (J.O.M.-B.); lizano@unam.mx (M.L.); 4 Departamento de Microbiología y Parasitología, Facultad de Medicina, Universidad Nacional Autónoma de México, Circuito Exterior S/N, Ciudad Universtiaria, Mexico City 04510, Mexico; padillaj@unam.mx; 5 Departamento de Medicina Genómica y Toxicología Ambiental, Instituto de Investigaciones Biomédicas, Universidad Nacional Autónoma de México, Circuito Exterior S/N, Ciudad Universitaria, Mexico City 04510, Mexico; 6 Centro de Investigación sobre Envejecimiento (CIE), Cinvestav, Unidad Sede Sur. Calzada de los Tenorios No. 235 Col. Granjas Coapa, Tlalpan, Mexico City 14400, Mexico

**Keywords:** mast cells, lysophosphatidylinositol, GPR55, CB2, chemotaxis, VEGF, cannabinoids, cancer

## Abstract

Mast cells (MCs) are the main participants in the control of immune reactions associated with inflammation, allergies, defense against pathogens, and tumor growth. Bioactive lipids are lipophilic compounds able to modulate MC activation. Here, we explored some of the effects of the bioactive lipid lysophosphatidylinositol (LPI) on MCs. Utilizing murine bone marrow-derived mast cells (BMMCs), we found that LPI did not cause degranulation, but slightly increased FcεRI-dependent β-hexosaminidase release. However, LPI induced strong chemotaxis together with changes in LIM kinase (LIMK) and cofilin phosphorylation. LPI also promoted modifications to actin cytoskeleton dynamics that were detected by an increase in cell size and interruptions in the continuity of the cortical actin ring. The chemotaxis and cortical actin ring changes were dependent on GPR55 receptor activation, since the specific agonist O1602 mimicked the effects of LPI and the selective antagonist ML193 prevented them. The LPI and O1602-dependent stimulation of BMMC also led to VEGF, TNF, IL-1α, and IL-1β mRNA accumulation, but, in contrast with chemotaxis-related processes, the effects on cytokine transcription were dependent on GPR55 and cannabinoid (CB) 2 receptors, since they were sensitive to ML193 and to the specific CB2 receptor antagonist AM630. Remarkably, GPR55-dependent BMMC chemotaxis was observed towards conditioned media from distinct mouse and human cancer cells. Our data suggest that LPI induces the chemotaxis of MCs and leads to cytokine production in MC in vitro with the differential participation of GPR55 and CB2 receptors. These effects could play a significant role in the recruitment of MCs to tumors and the production of MC-derived pro-angiogenic factors in the tumor microenvironment.

## 1. Introduction

Mast cells (MCs) are central players in distinct protective and deleterious inflammatory reactions triggered by innate and adaptive immunity [1,2]. In adults, they derive from bone marrow precursors that are recruited to all vascularized tissues where they finish their differentiation under the influence of microenvironmental conditions and locally produced mediators [3]. The differentiation of MCs is accompanied by morphological changes and the formation of numerous secretory granules that store preformed inflammatory substances, such as heparan sulphate, histamine, proteases, and cytokines. In addition, specific receptors are expressed in their plasma membrane, making them able to attend distinct stimuli, mounting different responses that range from chemotaxis and piecemeal degranulation to the extensive release of granule content (anaphylactic degranulation), as well as the synthesis of arachidonic acid derivatives and the long-term production of regulatory, pro-inflammatory, and pro-angiogenic cytokines [4]. 

Since MC recruitment has been identified as a hallmark in distinct chronically inflamed tissues, and the production of MC mediators is associated with accelerated growth rate and angiogenesis in some malignant tumors [5,6,7], the study of the factors that promote MC chemotaxis and the synthesis of pro-angiogenic molecules in this cell type is an active and relevant research field. 

Bioactive lipids such as sphingosine 1 phosphate (S1P), cannabinoids (CB), and lysophospholipids exert numerous regulatory functions in the immune system. Lysophosphatidylinositols (LPIs) are subspecies of lysophospholipids that contain inositol as its head group. The compounds 1-stearoyl- and 2-arachidonoyl LPI are formed by the action of PLA2 or PLA1, respectively [8,9]. In particular, the bioactive lipid 2-arachidonoyl-lysophosphatidylinositol (from now referred as LPI) has emerged as an important molecule involved in the spread of cancer and the recruitment of distinct immune cell types to sites of inflammation and tumor growth [10,11]. 

LPI is recognized by distinct G protein-coupled receptors (GPCRs), such as GPR18, GPR119, and GPR55 [12]. Of these, GPR55 is the best characterized to date [13]. Although it was considered an atypical cannabinoid (CB) receptor, now it is known that it belongs to the non-Edg P2Y receptor GPCR family and it is coupled to Gα_12/13_ or Gα_i/o_ in distinct cell types [8,12]. In humans, it has been mapped on chromosome 2 and is expressed in several tissues such as some brain regions, adrenals, jejunum, and spleen. GPR55 is also expressed in immune cells such as monocytes and NK cells [14]. 

LPI is secreted by distinct tumor cells [15,16] and its levels are significantly increased in patients with certain types of cancer [17]. On the other hand, LPI promotes the chemotaxis of neutrophils [18] and induces the production of pro-inflammatory cytokines in macrophages [19], suggesting that this lipid could be an important molecule involved in the communication between cancerous and immune cells. 

The aim of this work was to analyze, in vitro, the effect of LPI on MCs. Utilizing distinct approaches, such as Boyden’s chamber chemotaxis assays, Western blot, RT-PCR, and confocal microscopy, along with distinct pharmacological treatments in bone marrow-derived mast cells (BMMCs), we found that LPI induces significant effects on MC actin cytoskeleton remodeling, cell migration, and cytokine mRNA expression through the differential activation of GPR55 and the cannabinoid 2 (CB2) receptors. 

## 2. Results

### 2.1. Lysophosphatidylinositol Induces MC Chemotaxis and Activation of the LIMK/Cofilin Axis

**Figure 1:**
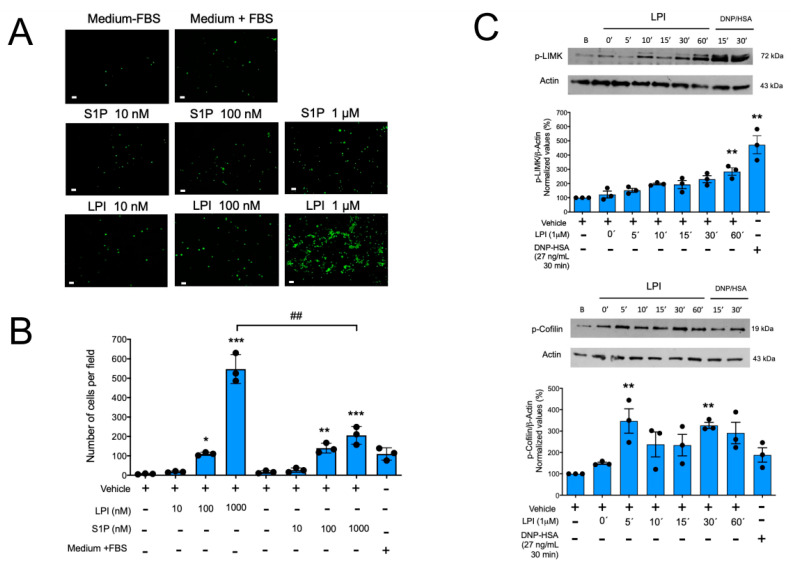
LPI promotes chemotaxis and changes on the LIMK/cofilin axis in MCs. Panel (**A**): representative image of a filter obtained in a migration assay of BMMCs towards distinct chemoattractants in the Boyden’s chamber. Scale Bar = 20 μm. Panel (**B**): quantification of migration of MCs towards sphingosine 1 phosphate (S1P) and lysophosphatidylinositol (LPI) is shown. Chemoattractants were dissolved first in a proper vehicle and then in serum-free media (Materials and Methods). Data are presented as the mean ± SEM of the number of cells observed in at least four microscope fields obtained with the 10× objective, from three independent experiments performed with distinct cell cultures, * *p* < 0.05; ** *p* < 0.01; *** *p* < 0.001 vs. vehicle; ## *p* < 0.01 vs. LPI. Panel (**C**): two million BMMCs were stimulated with 1 μM LPI during the indicated times, and then lysed to detect phosphorylation of LIMK and cofilin proteins by Western blot with specific antibodies. A representative gel image and the quantification of band intensity observed in at least three independent experiments are shown. ** *p* < 0.01; *** *p* < 0.001 vs. vehicle.

Aiming to characterize the effects of LPI in MCs, we decided to start with the evaluation of the capacity of this lipid to regulate degranulation in MCs. Mature, IgE-sensitized BMMCs were incubated with distinct concentrations of LPI alone or in the presence of two concentrations of the antigen DNP-HSA to determine the extent of cell degranulation. As observed in Supplementary Figure S1, LPI alone (tested at 100 nM or 1 μM) did not induce the secretion of the enzyme β-hexosaminidase, a preformed (granule-stored) mediator, although it was able to slightly enhance (10–15%) FcεRI-dependent degranulation. With the aim of testing whether LPI could be a chemoattractant to BMMCs, we decided to evaluate the migration of these cells to LPI utilizing Boyden’s chamber assays. As observed from Figure 1, panels A and B, LPI produced a significant concentration-dependent migration of BMMCs, and remarkably, this effect was higher than that observed with the well-characterized chemoattractant sphingosine 1-phosphate (S1P). 

Since LIMK and cofilin are the main regulators of actin cytoskeleton dynamics as they mediate the essential steps in the actin depolymerization/polymerization cycle necessary for cell movement, we analyzed the capacity of LPI to induce changes in their phosphorylation. BMMCs were stimulated with LPI, and the phosphorylation of LIMK and cofilin was determined by Western blot. As can be observed from Figure 1C, LPI stimulation caused a very rapid change in cofilin phosphorylation and the subsequent detectable phosphorylation of LIMK. Although rapid changes in the total amount of LIMK and cofilin cannot be ruled out, these results suggest that the LPI-induced chemotaxis of BMMCs is due to the activation of the canonical LIMK/cofilin axis. 

### 2.2. LPI Induces Changes in Actin Cytoskeleton Dynamics in MCs

**Figure 2:**
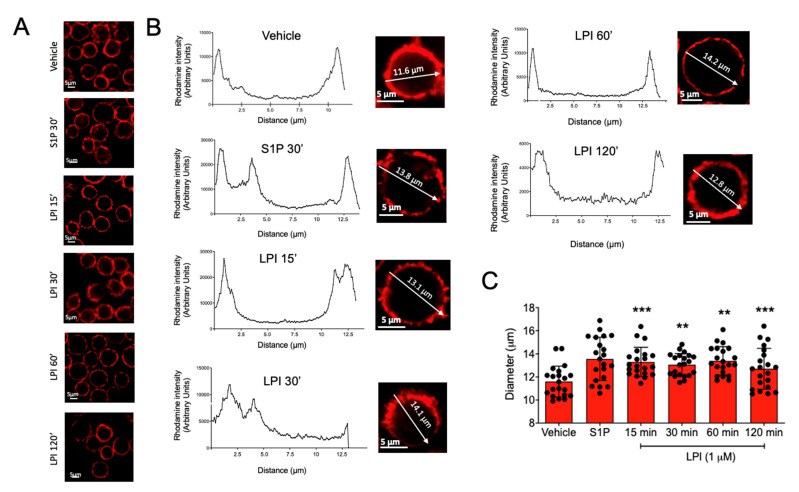
LPI-induced changes in cell size and actin ring polymerization in MCs. Panel (**A**): representative images of BMMCs stained with rhodamine-labeled phalloidin after treatments with vehicle, S1P (1 μM), and LPI (1 μM) at distinct times. Panel (**B**): representative images and analysis of cell size and the polymerization of the actin ring of BMMCs treated with S1P and LPI at distinct times. Utilizing the confocal microscopy software Zen 2.3 SP1 Blue Edition, a line was traced crossing at the equatorial plane of the individual cells and fluorescence values along the line were collected to calculate cell size using the graphs shown. Panel (**C**): quantification of changes in cell size induced by LPI in BMMC. Data are presented as the mean ± SEM of at least 20 cells analyzed under each condition; ** *p* < 0.01; *** *p* < 0.001 vs. vehicle.

Mast cell migration involves distinct steps that require actin polymerization and changes in cell shape and size [20,21]. With the aim of closely analyzing whether LPI could induce changes in cell actin cytoskeleton organization, we visualized changes in the size and the cortical actin ring induced by that bioactive lipid in BMMCs. Cells were incubated for distinct times with 1 μM LPI, and polymerized actin was detected utilizing phalloidin coupled to rhodamine. Analysis with confocal microscopy was performed and the cell size was measured by the distribution of fluorescence along a line traced in the equatorial plane of the cells (diameter). As can be observed from Figure 2, the addition of LPI or S1P caused a rapid increase in the cell size, since the mean diameter of the BMMCs changed from 11.5 ± 0.3 μm under vehicle-treated conditions to 13.4 ± 0.5 μm in the presence of LPI. Besides the changes in the cell size, cortical actin ring discontinuities were also observed. 

**Figure 3:**
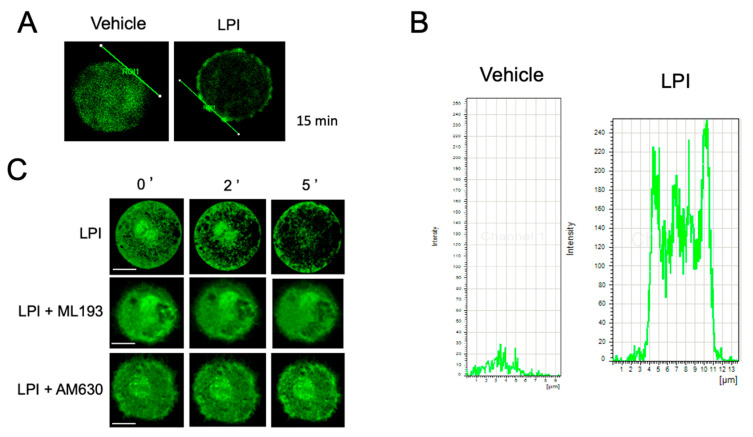
LPI induces actin cytoskeletal rearrangements and actin ring depolymerization in MCs. EGFP-BMMCs were incubated under distinct conditions before being processed for confocal microscopy. Panels (**A**,**B**): representative image of EGFP-BMMCs treated for 15 min with vehicle or LPI (1 μM). A line was traced on the edge of the cell and fluorescence values were obtained along the line to obtain representative graphs. Panel (**C**): EGFP-BMMCs were incubated in the presence of vehicle or LPI (1 μM) directly in a plate placed at the confocal microscope (Materials and Methods section). Images were taken every second for 300 s and representative images of a cell, obtained at time zero and distinct times after stimulation, are shown. When needed, cells were treated for 15 min with antagonists ML193 (100 nM) or AM630 (100 nM) before LPI addition. Data are representative of at least 10 cells analyzed under each condition, utilizing at least three independent cell cultures. Scale bar = 5 μm.

With the aim of closely analyzing the changes in the actin dynamics induced by LPI, we decided to generate BMMCs from C57BL6/J-Tg (CAG-EGFP) mice that express actin monomers tagged with the enhanced green fluorescent protein (EGFP) due to a targeted gene fusion (EGFP-BMMCs, Materials and Methods section). These cells were incubated in the presence of LPI and the continuity of the actin ring was analyzed by confocal microscopy. As can be observed from Figure 3A,B, LPI caused an observable discontinuity of the cortical actin ring. Moreover, EGFP-BMMCs were utilized to directly observe the changes in the actin cytoskeleton in real time. As shown in Figure 3C and Supplementary Video S1, LPI stimulation triggered an important re-organization of the actin cytoskeleton. Under basal conditions, the actin filaments were found distributed in the cytoplasm and nuclear membrane, and an actin mesh also surrounded secretory granules. However, after 2 min of LPI addition, the actin fibers rapidly distributed to the perinuclear zone and the total amount of cytoplasmic actin diminished. After 5 min of LPI addition, the perinuclear actin diminished and the actin fibers concentrated at the plasma membrane, surrounding the secretory granules. On the other hand, under basal conditions, the granules were homogeneously distributed in the cytoplasm and after 2 min of LPI addition, the granules seemed to fuse with one another. After 5 min, numerous granules were translocated to the plasma membrane and discontinuities in the actin ring appeared to coincide with granules that seemed to be fused with the plasma membrane. 

### 2.3. LPI-Induced Chemotaxis Depends on the Activation of GPR55 Receptor

**Figure 4:**
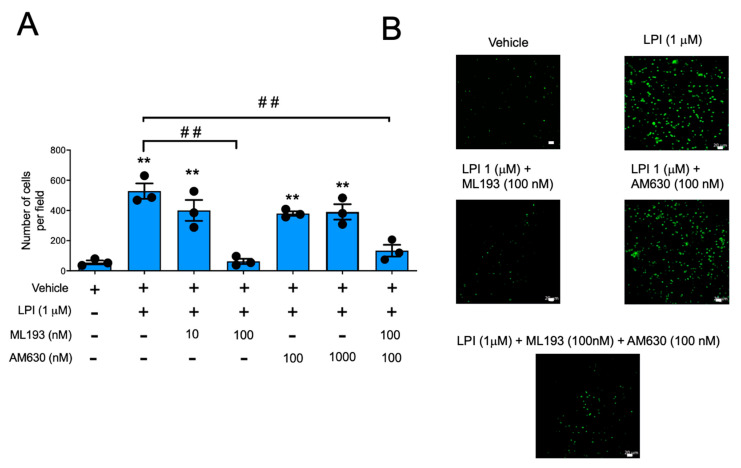
LPI-induced chemotaxis is mediated by GPR55, but not CB2 receptors in BMMCs. Panel (**A**): BMMCs were placed in the Boyden’s chamber to quantify migration towards LPI in the presence of ML193, AM630, or a combination of both antagonists. Data in graph are presented as the mean ± SEM of at least three independent experiments performed with distinct cell cultures; ** *p* < 0.01 vs. vehicle; ## *p* < 0.01 vs. LPI. Panel (**B**): representative image of a Boyden’s chamber experiment utilizing LPI (1 μM) alone or in the presence of ML193 (100 nM), AM630 (100 nM), or a combination of both antagonists (100 nM ML193 + 100 nM AM630). No significant difference was observed when the effect of ML193 alone was compared with the ML193 + AM630 group (*p* = 0.3704). Scale Bar = 20 μm.

LPI is a bioactive lipid able to activate distinct GPCRs. Among them, GPR55, together with the CB1 and CB2 receptors, have been detected in MCs [22,23], with CB2 and GPR55 being the most abundantly expressed [23,24]. With the objective of identifying the receptor involved in the effects of LPI, we decided to utilize selective antagonists of GPR55 and CB2 receptors. First, the effect of pre-incubation with the GPR55-specific antagonist ML193 or the CB2-specific antagonist AM630 on LPI-induced polymerization of the actin ring was evaluated using confocal microscopy. Figure 3C and Supplementary Video S2 show that the addition of ML193 (100 nM) prevented the effects of LPI on actin polymerization in EGFP-BMMCs. In contrast, the addition of AM630 (100 nM) caused a brief delay in the engrossing of the actin ring and prevented actin depolymerization in the perinuclear area (Supplemental Video S3 ). When the effect of the mentioned antagonists was tested in Boyden’s chamber experiments, LPI-induced migration was efficiently inhibited with ML193 and with the combination of ML193 and AM630, but not with AM630 alone (Figure 4). 

### 2.4. Specific GPR55 Agonist O-1602 Promotes Chemotaxis and Activation of LIMK in BMMCs 

**Figure 5:**
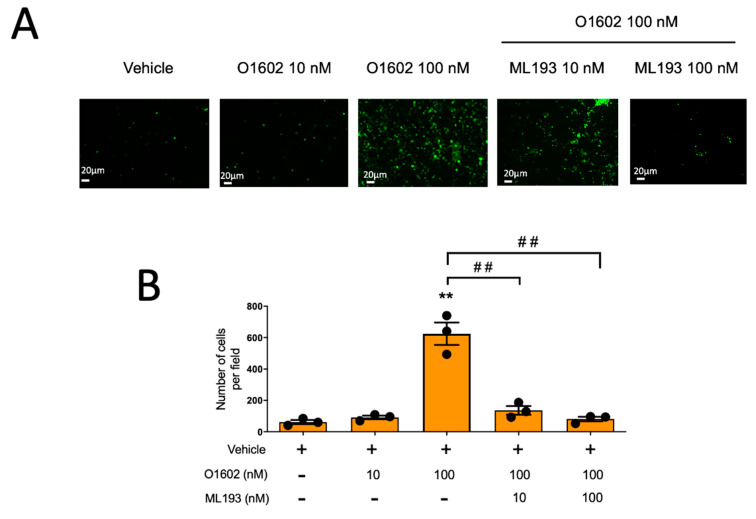
GPR55-specific agonist O1602 causes GPR55-dependent migration in BMMCs. Panel (**A**): representative image of a filter obtained in a migration assay of BMMCs towards distinct concentrations of O1602 alone or in the presence of ML193. Panel (**B**): quantification of cell migration of BMMCs towards O1602; ** *p* < 0.01 vs. vehicle; ## *p* < 0.01 vs. O1602.

To further support the role of GPR55 on the chemotaxis of MCs, we tested the effect of the GPR55-specific agonist O-1602 on the migration of BMMCs. As observed with LPI, O1602, at concentrations ranging from 1 nM to 1 μM, was unable to induce the anaphylactic degranulation of MCs. However, this compound exerted a significant chemoattractant activity on BMMCs. As shown in Figure 5, O1602 induced cell migration and the addition of ML193 fully prevented BMMC chemotaxis to this compound. 

### 2.5. GPR55 Receptor Mediates the Chemotaxis of BMMCs to Conditioned Media from Distinct Mouse and Human Cancer Cells

**Figure 6:**
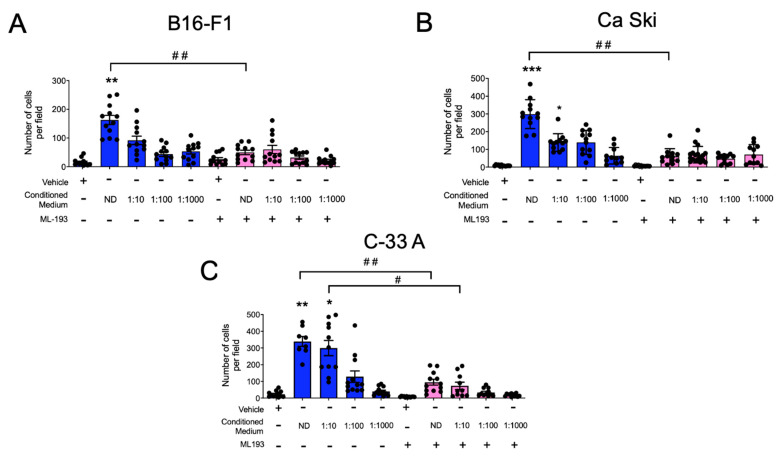
BMMCs migrate towards cancer cell-conditioned media in a GPR55 receptor-dependent fashion. Conditioned media from (**A**) the B16-F1 murine malignant melanoma cell line as well as (**B**) Ca Ski and (**C**) C-33 A human cervical cancer cell lines were collected and diluted as described in the Materials and Methods section. Boyden’s chamber chemotaxis experiments were performed utilizing BMMCs in the presence of vehicle or ML193 (100 nM). Data are presented as the mean ± SEM of at least four microscope fields observed with the 10X objective obtained from at least three independent experiments. One way ANOVA vs. vehicle (without ML193) values, * *p* < 0.05; ** *p* < 0.01; *** *p* < 0.001 vs. vehicle; # *p* < 0.05; ## *p* < 0.01 vs. ML193 non-treated groups. ND, non-diluted conditioned media. Blue and pink bars show values obtained in the absence or presence of the GPR55 antagonist ML-193, respectively.

Aiming to test whether the GPR55-mediated migration of MCs could be involved in their recruitment to malignant tumors, we evaluated the chemotaxis of BMMCs towards conditioned media from distinct murine and human cancer cell lines. First, conditioned media from cultures of B16-F1, a murine malignant melanoma cell line, were collected as described in the Materials and Methods section, and chemotaxis assays towards serial dilutions of this media, alone or in the presence of ML193, were determined. Figure 6A shows that BMMCs migrated to the conditioned media of B16-F1 cells, and the ML193 compound prevented this cell migration. Then, we evaluated BMMC migration towards conditioned media from human transformed cells. Conditioned media obtained from the human cervical carcinoma cell lines Ca Ski and C-33 A were obtained and used for chemotaxis experiments. As can be observed in Figure 6B,C, the BMMCs migrated to transformed cell-conditioned media with distinct intensities. Ca Ski-conditioned media induced the migration of BMMCs, and this phenomenon was sensitive to ML193. Interestingly, C-33 A-conditioned media promoted higher values of cell migration that was also sensitive to the GPR55 antagonist. Together, our data show that BMMCs migrate towards compounds produced by cancer cell lines with an important participation of the GPR55 receptor, whose recognized ligand is LPI. The data also suggest that cervix cancer cells, as for other cell lines derived from human tumors [15,16], release bioactive lipids. 

### 2.6. LPI and O1602 Promote Cytokine mRNA Accumulation with the Participation of GPR55 and CB2 Receptors

**Figure 7:**
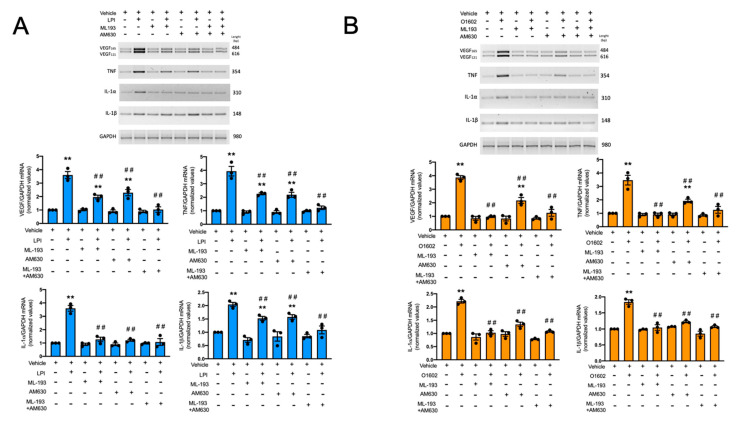
GPR55 and CB2 receptors mediate LPI and O1602-induced cytokine mRNA accumulation in BMMCs. Panel (**A**): BMMCs were treated for three hours with LPI (1 μM) in the presence of vehicle, ML193 (100 nM), or AM630 (100 nM). Total RNA extraction, cDNA synthesis, and RT-PCR were performed as described in the Materials and Methods section. A representative image of a 2% agarose gel with the corresponding amplification fragments is shown in the upper part of the figure, whereas, in the lower part, the quantification of at least three experiments is shown. Panel (**B**): cells were treated with O1602 (100 nM) and the respective GPR55 or CB2 receptor antagonists, as in panel A. In both panels, the effect of each antagonist alone and the combination of both is also shown. Data are presented as the mean ± SEM of at least three independent experiments. ** *p* < 0.01 vs. vehicle; ## *p* < 0.01 vs. LPI or O1602. Blue and orange bars show data obtained stimulating the cells with LPI or with the GPR55 agonist O1602, respectively.

The physiological functions of MCs require a series of distinct events that lead to the recruitment of their cell precursors to specific locations, cell maturation in situ, and the production of distinct immune mediators that control inflammation, angiogenesis, and tissue repair, among other processes. To characterize the effect of LPI and its receptors on the production of active mediators by MC, we decided to analyze the synthesis of distinct cytokine mRNAs in response to LPI in BMMC by semi-quantitative RT-PCR. Figure 7 shows that LPI promoted an increase in the mRNA synthesis of vascular endothelial growth factor (VEGF), tumor necrosis factor (TNF), and the pro-inflammatory cytokines IL-1α and IL-1β. The cells were exposed for 3 h to 1 μM LPI or 100 nM O1602 in the presence or absence of 100 nM ML193 or 100 nM AM630. Afterwards, the cells were harvested and the total mRNA was extracted to synthesize cDNA and amplify the mentioned gene sequences. As illustrated in Figure 7A, LPI promoted the mRNA synthesis of VEGF, TNF, IL-1α, and IL-1β. As expected, ML193 prevented the LPI-induced accumulation of these mRNAs, but, unexpectedly, the inhibition of VEGF, TNF, and IL-1β mRNA accumulation was only close to 50%. In contrast, the accumulation of IL-1α mRNA was fully sensitive to ML193. To test whether residual mRNA synthesis could be dependent on the activation of other receptors, the CB2 antagonist AM630 was utilized. As can be observed in Figure 7A, AM630 also partially inhibited the LPI-dependent accumulation of VEGF, TNF, and IL-1β mRNAs, and only the combination of both antagonists fully prevented the effects of LPI. Of note, IL-1α accumulation was fully sensitive to AM630. These data suggest that LPI promotes the production of pro-angiogenic and pro-inflammatory mediators in MCs and that, in contrast with chemotaxis, this effect requires the participation of both GPR55 and CB2 receptors. To corroborate this finding, the cells were stimulated with O1602, and cytokine mRNA synthesis was also analyzed. Figure 7B shows that, as in the case of LPI, the GPR55 agonist provoked the synthesis of VEGF, TNF, IL-1α, and IL-1β mRNAs. These effects were, as expected, fully sensitive to ML193. However, O1602’s effects on VEGF and TNF mRNA accumulation were also partially sensitive to the CB2 agonist AM630, since around 50% of the response was observed in the presence of that compound. The accumulation of IL-1α and IL-1β mRNAs was fully sensitive to AM630. These data suggest that GPR55 fully mediates the cytokine mRNA accumulation in response to O1602, but also supports the idea that the CB2 receptor plays a role in the GPR55-induced cytokine synthesis in BMMCs. 

## 3. Discussion

Bioactive lipids are important regulators of cell functions in physiological and pathological conditions [9]. Among them, LPI has been identified as a novel molecule produced during inflammatory reactions and cancer progression. LPI has shown significant effects on the promotion of cancer cell proliferation and migration [10], but its effects on immune cells, particularly in MCs, are not fully described. The main findings of this work are: (1) LPI does not induce anaphylactic degranulation, but enhances FcεRI-dependent β-hexosaminidase release and strongly promotes the chemotaxis of BMMCs; (2) LPI treatment leads to significant reorganization of the actin cytoskeleton, which is observable as an increase in cell size and the formation of depolymerized cortical actin zones; (3) the effects of LPI on actin polymerization and chemotaxis are dependent on GPR55 receptor activation; (4) BMMCs migrate towards conditioned media obtained from murine and human transformed cell lines in a GPR55-dependent fashion; and (5) LPI and O1602 promote cytokine mRNA expression with the participation of both GPR55 and CB2 receptors. 

Anaphylactic degranulation, characterized by the rapid release of preformed granule content, is a marker of full MC activation. In our hands, LPI was not able to induce degranulation, but increased the secretion of β-hexosaminidase when added shortly before IgE/Ag. The signal transduction system of the FcεRI couples the recognition of IgE/Ag complexes to the secretion of pro-inflammatory and regulatory mediators by activating the molecules involved in cytoplasmic changes, calcium mobilization, and cytokine gene expression, but also activates intracellular enzymes involved in the formation of some active lipids, such as S1P, that enhances degranulation in an autocrine fashion [25]. Our results showing that LPI causes an increase in FcεRI-induced degranulation suggest that this compound should be further studied as a member of the bioactive lipids able to modify anaphylactic reactions, probably due to its effects on actin cytoskeleton organization. If this lipid is synthesized after the IgE/Ag stimulation of MCs (as S1P) remains an open question [26]. 

The reorganization of the actin cytoskeleton occurs during the migration of MCs to distinct chemokines and also occurs during the secretion of mediators from this cell type [20,21,27]. Data from Western blots suggest changes in LIMK and cofilin phosphorylation, although differences in the total amount of these proteins cannot be ruled out. To our knowledge, this is the first time that changes in the actin cytoskeletal dynamics in response to LPI have been reported in MCs. However, these results are in line with those obtained with distinct chemoattractants and secretagogues for MCs. For example, an increase in cell size has been observed in the RBL-2H3 cell line after treatment with the chemotactic cytokine IL-8 [27]. On the other hand, the presence of discontinuities in the cortical actin ring has been associated with a secretory actin phenotype in MCs [27]. Our closer analysis of actin polymerization utilizing BMMCs derived from EGFP-expressing mice showed that LPI induces rapid rearrangements of actin cytoskeleton that, in the first minutes, resemble the migratory actin phenotype (characterized by perinuclear actin clusters and discontinuities in the cortical actin ring). However, a few minutes later, the actin cytoskeleton resembles a secretory actin phenotype, with low perinuclear actin signal and a discontinuous actin ring. 

The confocal images suggest that LPI induces actin reorganization to promote migration, but also indicate that it promotes the secretion of granule content. Remarkably, no anaphylactic degranulation was observed in MCs treated with LPI alone, suggesting that LPI promotes the secretion of mediators through a mechanism of compound exocytosis or piecemeal degranulation. On the other hand, LPI increases the secretion of β-hexosaminidase in response to IgE/Ag complexes, suggesting that the actin reorganization promoted by LPI facilitates the secretory process. In analyzing the response of distinct preparations of MCs, it has been observed that the secretory actin phenotype abrogates the migratory phenotype in conditions when a chemoattractant precedes to a degranulation inducer [21,27]. In the light of this model, our results suggest that LPI initially promotes the migration of MCs and, later, with the participation of other stimuli, the secretion of mediators. This could be relevant to the recruitment of MCs to tumors, since after being attracted to tumor mass, MCs might release mediators to increase angiogenesis or exert other functions. 

Our results show, for the first time, that BMMCs strongly migrate to the conditioned media of transformed cell lines in a GPR55 fashion. In this regard, it has been shown that LPI is secreted by distinct malignant cell lines, such as the ovarian cancer cells OVCAR-3, OVCAR-5, and COV-362 [15], being able to induce the migration and proliferation of human endothelial colony-forming cells (ECFCs) [15]. Moreover, prostate cancer cells (PC-3 cell line) have been demonstrated to synthesize LPI by the cytosolic phospholipase A2 (PLA2). In these cells, LPI is secreted through the ATP-binding cassette transporter ABCC1/MRP1 and activate GPR55 in an autocrine loop [16]. In the same line, indirect evidence suggests the production of LPI by cancer cells in vivo, since elevated LPI plasma levels have been found in breast cancer patients [28] and ovarian cancer patients [17] when compared to healthy individuals. Our observation of the migration of MCs towards conditioned media from transformed cell lines strongly suggests a role of LPI (or other GPR55 ligands) in the chemotaxis of MCs to tumor niches. The presence of MCs in distinct malignant tumor types has been widely reported [5]. In these locations, they exert pro-inflammatory and pro-angiogenic actions, but also seem to coordinate protective responses [5,6,29].

Distinct molecules have been described as MC chemoattractants [30], and some of them have been proposed to be involved in the recruitment of this cell type to zones of tumor growth. For example, MCs that migrate towards conditioned media form distinct tumor cell lines in vitro [31,32], and Stem Cell Factor (SCF) has been proven to induce MC infiltration in tumors in vivo [33]. The chemokine CCL5 has been also proposed to participate in the incorporation of MCs to tumors, since it was observed expressed in smooth muscle tumor cells and tumor-infiltrating MCs express the CCR3 receptor [34]. On the other hand, CXCL12 is produced by several solid tumors and tumor-associated MCs express CXCR4 [35,36]. In the case of lipid mediators, it has been shown that prostaglandin E2 induces MC migration and MC accumulation to sites where it was administered in vivo [37]. Our results showing BMMC migration towards cervix cancer cells led to the hypothesis that the LPI/GPR55 axis could play a role in the incorporation of MCs in that type of cancer. This possibility should be further explored, since an important increase in tryptase-positive MCs has been detected in samples of invasive cervix carcinoma, but not in cervix tissue derived from healthy women [38]. Together, our results provide evidence indicating that LPI and its receptor GPR55 could participate in the incorporation of MCs to sites of tumor growth and that GPR55 blockage could be considered a therapeutic strategy to inhibit the recruitment of MCs to solid tumors. 

Notably, LPI promoted the synthesis of VEGF and TNF mRNAs with the involvement of the GPR55 receptor in BMMCs. The results shown are in line with those previously reported, indicating that this bioactive lipid can induce gene transcription through the activation of MAPK (such as p38) and transcription factors such as ATF2 [39] and NFAT [40]. The complete signal transduction pathway activated by GPR55 in MCs should be further investigated to evaluate the role of p38 MAPK or other proteins on the control of cytokine expression in this cell type. On the other hand, VEGF and TNF mRNA accumulation is also regulated by post-transcriptional events, such as mRNA stabilization [41,42], and an impact of LPI on the activation of mRNA-stabilizing factors in the synthesis of pro-inflammatory and pro-angiogenic mediators cannot be ruled out. LPI-dependent cytokine mRNA accumulation was also partially sensitive to the CB2-specific antagonist AM630, suggesting the participation of CB2 receptors on LPI actions. Moreover, partial inhibitory effects of AM630 on the synthesis of VEGF and TNF mRNAs caused by O1602 strongly support the participation of CB2 receptors on the actions of GPR55 in BMMCs. The data suggest that, besides the actions of GPR55 and CB2 receptors alone, the formation of GPR55-CB2 dimers could participate in the expression of cytokine genes triggered by LPI. GPR55 receptor interaction with distinct GPCRs, such as the S1PR [43] and the lysophosphatidic acid (LPA) 2 receptor [44], has been demonstrated in some cell preparations. Specifically, GPR55 dimer formation with cannabinoid receptors has been previously observed in neutrophils, transfected HEK293 cells, and cancer cells [18,45,46]. In MCs, GPR55-CB2 receptor dimerization has been also suggested, since LPI and the specific CB2 agonist HU308 inhibited FcεRI-dependent degranulation in BMMCs, and these actions were prevented by AM630 [24]. The participation of monomeric and heterodimeric GPR55 and CB2 receptors on LPI and O1602 effects on cytokine mRNA accumulation in MCs is an open possibility that should be further explored. 

Our data show important chemotactic effects of LPI on a cell culture that renders cells closely resembling mucosal MCs [47]. Although MCs with a connective tissue phenotype (i.e., expressing tryptase) have been found to be associated with tumors and tumor-associated MCs seem to suffer important changes to their phenotype due to conditions prevalent in the tumor environment [29], mucosal-type MCs have been importantly related to colon cancer [48] and to adenoma–carcinoma progression [49]. Future research should be conducted to analyze the effects that the LPI/GPR55 axis exerts on connective tissue MCs and whether this is related to the pro- and anti-tumoral roles that have been described for MCs. 

**Figure 8:**
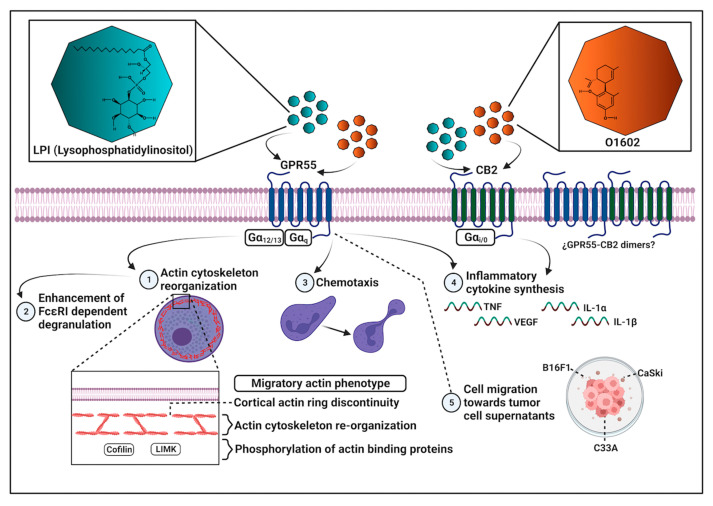
Overview of main LPI effects on chemotaxis, cytoskeletal rearrangements, and cytokine mRNA production in BMMCs. LPI promotes chemotaxis and changes in the actin cytoskeleton dynamics in BMMCs through GPR55 receptor activation, whereas its actions on cytokine mRNA accumulation partially require the participation of CB2 receptors. LPI also increases degranulation triggered by FcεRI. The GPR55 receptor is involved in BMMC migration to tumor cell-conditioned media. GPR55 agonist O1602 promotes chemotaxis and cytokine mRNA accumulation, but the latter effect is dependent on CB2 receptors.

The obtained results point to the complex participation of GPR55 and CB2 receptors on the effects of LPI on BMMCs, since some responses, such as chemotaxis and changes to the actin cytoskeleton dynamics, seem to depend only on GPR55 activation, while cytokine mRNA accumulation requires both GPR55 and CB2 receptors. On the other hand, GPR55 emerges as a central molecule mediating LPI-induced migration and actin cytoskeleton remodeling in MCs, which places that receptor as an attractive target to inhibit the recruitment of MCs to tumor niches. The main findings obtained in this work are schematized in Figure 8. 

## 4. Materials and Methods

### 4.1. Mice

Wild-type C57BL/6J and C57BL6/J-Tg (CAG-EGFP) 1310sb/LeySopJ mice (stock numbers 000664 and 006567, respectively) were purchased from The Jackson Laboratory (Bar Harbor, ME, USA) and maintained in the Unit for Production and Laboratory Animal Experimentation (UPEAL) from Cinvestav. A breeding pair of EGFP-expressing mice to generate the EGFP-BMMCs used in this paper was generously donated by Dr. José Vazquez-Prado, from the Pharmacology Department (Cinvestav, Zacatenco Campus). The animals were maintained under controlled standard humidity and temperature (22–24 °C) conditions, with ad libitum access to food and water and a 12 h light/12 h dark cycle. All procedures were performed according to our Institutional Committee for the Care and Use of Laboratory Animals (CICUAL), under the approved protocol 137-15. The authorized protocol follows the National Institutes of Health (NIH) guidelines for the use and care of laboratory animals and the Official Mexican Norm NOM-062-ZOO-1999.

### 4.2. Reagents and Antibodies

Monoclonal IgE (clone SPE7), RPMI 1640, 2-mercaptoethanol (2-ME), Laemmli buffer, L-α-lysophosphatidylinositol (LPI), and sphingosine-1-phosphate (S1P) were purchased from Sigma-Aldrich (St. Louis, MO, USA). HEPES, non-essential amino acids (NEAA), penicillin, streptomycin, and fetal bovine serum (FBS) were obtained from Gibco-BRL-Life Technologies (Gaithersburg, MD, USA). Interleukin (IL)-3 was purchased from PeproTech (Rocky Hill, NJ, USA). The GPR55 synthetic agonist, O-1602, was purchased from Cayman Chemical (Ann Arbor, MI, USA), while the GPR55 antagonist, ML-193, was purchased from TOCRIS (Bristol, UK). The CB2 antagonist, AM630, was from Sigma-Aldrich (St. Louis, MO, USA). Anti-p-cofilin (pSer 3) antibodies were obtained from Cell Signaling Technology (Danvers, MA, USA). Anti-p-LIMK (pThr 508/505) and anti-β-actin (C-terminal) antibodies were purchased from Santa Cruz Biotechnology (Dallas, TX, USA). The secondary antibodies, HRP-coupled anti-mouse and anti-rabbit, were obtained from Jackson ImmunoResearch (West Grove, PA, USA). Rhodamine-labeled phalloidin and calcein-AM were obtained from Life Technologies (Carlsbad, CA, USA). DAPI was purchased from Invitrogen (Carlsbad, CA, USA). The polycarbonate filters were from Neuro Probe, and bovine skin gelatin from Sigma-Aldrich (St. Louis, MO, USA)

### 4.3. Generation of BMMCs, IgE Sensitization and Determination of β-hexosaminidase Release

BMMCs were isolated as previously described [50]. Briefly, bone marrow (BM) was extracted from both tibias of 6- to 8-week-old mice. Freshly isolated BM was placed on media composed of RPMI-1640 supplemented with 20 ng/mL IL-3, 10% FBS, 25 mM HEPES buffer, 1 mM sodium pyruvate, 50 μM 2-Mercaptoethanol (ME), 100 IU/mL penicillin, 100 μg/mL streptomycin, and 1X non-essential amino acids (NEAA, from a 100X stock from Invitrogen). All components of the cell culture medium were purchased obtained from Gibco-BRL-Life Technologies (Gaithersburg, MD, USA). The cells were cultured at 37 °C under 5% CO_2_ atmosphere for 4 to 6 weeks, changing media once per week. The maturity of the BMMCs was evaluated by performing flow cytometry (FACS) to analyze the expression of the FcεRI receptor on cellular membrane and only 4–6-week-old cultures with more than 95% FcεRI-positive cells were utilized. Since it has been shown that monomeric IgE promotes the maturation of BMMCs, allowing them to better respond to distinct physiological stimuli [51,52], these cells were sensitized for 18 h with monomeric anti-dinitrophenol IgE (mIgE, 100 ng/mL, Sigma-Aldrich (St. Louis, MO, USA) at 37 °C before being used for all experiments. The functional status of BMMC was routinely evaluated by measuring the release of β-hexosaminidase in response to FcεRI triggering, as described previously [53].

### 4.4. Immunofluorescence and Confocal Microscopy

To analyze actin ring polymerization rearrangements, two strategies were used. For the first one, 2 × 10^6^ BMMCs were treated for different periods of time (15, 30, 60, and 120 min) with 1 μM LPI at 37 °C. As a control, the cells were treated for 30 min with 100 nM sphingosine-1-phosphate (S1P). After treatment, the BMMCs were resuspended in 150 μL of 1X phosphate buffered saline (PBS), and the cells were settled for 15 min on a positive-charged glass slide. Later, the cells were fixed for 15 min with 4% paraformaldehyde (PFA). The slide was washed three times with 1X PBS and, after treatment, the blocking solution (PBS 1X, BSA 10%, 50 μL Donkey serum and 1 μL Tween-20) was added for 2 h. To detect the actin ring, the cells were incubated overnight with rhodamine-labeled phalloidin (1:750) at 4 °C in the dark. The next day, the cells were washed for 20 min with 1X PBS, and DAPI (1:500) was added for 5 min to detect the cell nuclei. The cells were washed again, and the preparation was sealed with DABCO. Once finished, the slide was observed under a Zeiss Airyscan LSM-800 confocal microscope (Carl Zeiss, Oberkochen, Germany). The analysis of cell size and the distribution of fluorescence was performed with the software Zen 2.3 SP1 Blue Edition. The second strategy consisted of determining the changes in actin polymerization by the direct observation of BMMCs derived from EGFP-expressing mice using confocal microscopy. Briefly, 3 × 10^6^ EGFP-expressing BMMCs in RPMI medium were incubated overnight in a 35 mm Petri dish with a 23 mm glass base (Fluorodish, World Precision Instruments, WPI, Sarasota, FL, USA) at 37 °C in a CO_2_ incubator. The media was collected and cells were washed twice with PBS before the addition of 1 mL Tyrode buffer maintained at 37 °C. The Petri dishes were taken to the confocal microscope and stimulation with LPI alone or in the presence of antagonists was performed. Images were acquired with a 63X objective using a 4X optical zoom in a DMi8 Stellaris 5 confocal microscope and utilizing the software Leica Application Suite (LAS) X, version 4.3.0.24308. Supplemental videos were constructed with serial images taken every second for 5 min before being compressed into 18 s (Supplementary Videos S1 and S2) or 50 s (Supplementary Video S3).

### 4.5. Chemotaxis Assay

To determine whether LPI was a chemoattractant for MCs, and to identify the receptor involved, Boyden chamber modified assays were performed. A 48-well Boyden chamber was used (Neuro Probe, Gaithersburg, MD). Polycarbonate filters (25 × 80 mm) with 8 μm pore size were coated for 2 h with 2% of bovine skin gelatin (Sigma-Aldrich, St. Louis, MO, USA) at 37 °C and air dried. The BMMCs were washed with 1X PBS to remove the excess medium as FBS has been demonstrated to be a chemoattractant for MCs. Once washed, the cells were loaded for 1 h with 1 μg/L calcein-AM at 37 °C (Sigma-Aldrich (St. Louis, MO, USA). After loading, the cells were washed three times and resuspended in migration medium (MM, serum-free RPMI-1640, pH 7.3). Distinct ligands and antagonists (stocks dissolved in 1% DMSO solvents and diluted in MM) were added to the bottom wells in a 30 μL volume. In the upper wells, 40 × 10^3^ mature, IgE-sensitized BMMCs were placed. In all experiments with agonists and antagonists, the DMSO concentration was kept to less than 0.01%. As a negative control, MM with proper vehicle dilution was used, while medium RPMI supplemented with 10% FBS served as a positive control. In assays using ML-193 and AM630 antagonists, cells were pretreated for 15 min with different concentrations of these compounds at 37 °C before being placed on the chemotaxis chamber and the antagonists were also added to the bottom wells, mixed with LPI. The chamber was then incubated for 3 h in a humidified incubator at 37 °C with 5% CO_2_. Migrated cells adhering to the gelatinized filter were observed using a Zeiss LSM-800 Airyscan confocal microscope (Carl Zeiss, Oberkochen, Germany), using the software Zen 2.3 SP1 Blue Edition. Quantification was performed using Image J 1.53t software, analyzing four randomly selected fields obtained with a 10X objective per condition. The data are presented as the mean ± SEM of the number of cells observed in each field. 

### 4.6. Western Blot

Approximately 2 × 10^6^ cells per condition were resuspended in 1 mL of Tyrodes-BSA buffer (20 mM HEPES buffer at pH 7.4, 135 mM NaCl, 5 mM KCl, 1.8 mM CaCl_2_, 1 mM MgCl_2_, 5.6 mM Gly and 0.05% BSA). After stimulation, the cells were lysed on 1X Laemmli buffer and boiled for 15 min before being separated in SDS-PAGE. The proteins were transferred to polyvinylidene difluoride (PVDF) membranes in a semi-dry transfer unit. PVDF membranes were blocked for 2 h with 4% dry skimmed milk. The membranes were washed with TBS-T buffer (25 mM Tris, 0.9% NaCl, 0.05% Tween-20) and incubated overnight with the specific antibodies (anti-p-LIMK, anti-p-cofilin or anti-β-actin). The next day, the membranes were washed with TBS-T buffer and incubated for 1 h with the specific secondary antibody (1:5000) at room temperature. The membranes were washed again three times, and later visualized by chemiluminescence utilizing X-ray films. Densitometric analysis was performed with the software Molecular Imager Universal Hood II (Software Image Lab v.5.0, Bio-Rad, Hercules, CA, USA). 

### 4.7. Transformed Cell Culture and Collection of Conditioned Media

B16-F1 cells (ATCC stock number CRL-6323) were grown as described previously [54]. Briefly, cells maintained in frozen vials were thawed and grown in T75 flasks with low-glucose DMEM, 10% FBS, and standard concentrations of antimycotic and antibiotics, until 80% cell density was reached. Two days later, the conditioned media were collected and frozen until use. Cervical cancer-derived cell lines C33A and Ca Ski were purchased from ATCC (stock numbers HTB-31 and CRL-1550, respectively). For conditioned media collection, 2.5 × 10^6^ cells were seeded in 100 mm Petri dishes using RPMI and DMEM (Gibco, Waltham, MA, USA) supplemented with 10% FBS (Gibco) for Ca Ski and C33-A, respectively. After 24 h, the conditioned media was collected and centrifuged at 200× *g* for 5 min. To ensure the removal of cellular debris, the media was passed through a sterile 0.22 μm filter and stored at −70 °C until further use. For chemotaxis experiments, supernatants were diluted with the corresponding fresh media, and migration towards undiluted media was used as control.

### 4.8. RNA Extraction and RT-PCR

Approximately 2 × 10^6^ cells were stimulated as indicated, and then collected by centrifugation at 4 °C. The pellets were lysed by adding TRI-reagent (Sigma Aldrich, St. Louis, MO, USA), according to the manufacturer’s instructions. cDNA synthesis was performed using the RevertAid First Strand cDNA synthesis kit (Thermo Fischer Scientific, Waltham, MA, USA). The primers for RT-PCR are listed in Table 1, and amplification conditions were used as reported in the indicated references. The PCR products were analyzed using agarose gel (2%) electrophoresis, and the gels were stained using ethidium bromide. The gels were photographed using a MiniBIS Pro System (DNR Bio-Imaging Systems, Neve Yamin, Israel), utilizing the Gel Quant Express program. 

### 4.9. Statistical Analysis

The results are expressed as the mean ± SEM of at least three independent experiments performed with independent cell cultures. Some results were normalized considering basal (vehicle) or t = 0 conditions as 100% or 1. The data were analyzed by one-way analysis of variance (ANOVA), followed by Dunnett’s test. For some comparisons, an unpaired Student’s *t* test was utilized. All analyses were performed with GraphPad Prism v.5 (GraphPad Software, San Diego, CA, USA). 

## Figures and Tables

**Table 1:** Oligonucleotides utilized for semi-quantitative RT-PCR.

Cytokine	Forward	Reverse	Reference
VEGF	CTGCTCTCTTGGGTCCACTGG	CACCGCCTTGGCTTGTCACAT	[55]
TNF	TTCTGTCTACTGAACTTCGGGGTGATCGGTCC	GTATGAGATAGCAAATCGGCTTGTGGG	[56]
IL-1 alpha	CTCTAGAGCACATGTACAGAC	TGGAATCCAGGGGAAACACTG	[57]
IL-1 beta	TTGACGGACCCCAAAAGATG	AGAAGGTGCTCATGTCCTCA	[57]
GAPDH	TGAAGGTCGGTGTGAACGGATTTGGC	CATGTAGGCCATGAGGTCCACCAC	[58]

## Data Availability

Data are available upon request to corresponding author.

## Supplementary Materials

The following supporting information can be downloaded at: https://www.mdpi.com/article/10.3390/ijms24076316/s1.

## References

[1] Valent P., Akin C., Hartmann K., Nilsson G., Reiter A., Hermine O., Sotlar K., Sperr W.R., Escribano L., George T.I. (2020). Mast cells as a unique hematopoietic lineage and cell system: From Paul Ehrlich’s visions to precision medicine concepts. Theranostics.

[2] Galli S.J., Gaudenzio N., Tsai M. (2020). Mast Cells in Inflammation and Disease: Recent Progress and Ongoing Concerns. Annu. Rev. Immunol..

[3] St John A.L., Rathore A.P.S., Ginhoux F. (2023). New perspectives on the origins and heterogeneity of mast cells. Nat. Rev. Immunol..

[4] Mukai K., Tsai M., Saito H., Galli S.J. (2018). Mast cells as sources of cytokines, chemokines, and growth factors. Immunol. Rev..

[5] Varricchi G., Galdiero M.R., Loffredo S., Marone G., Iannone R., Marone G., Granata F. (2017). Are Mast Cells MASTers in Cancer?. Front. Immunol..

[6] Komi D.E.A., Redegeld F.A. (2020). Role of Mast Cells in Shaping the Tumor Microenvironment. Clin. Rev. Allergy Immunol..

[7] Lichterman J.N., Reddy S.M. (2021). Mast Cells: A New Frontier for Cancer Immunotherapy. Cells.

[8] Yamashita A., Oka S., Tanikawa T., Hayashi Y., Nemoto-Sasaki Y., Sugiura T. (2013). The actions and metabolism of lysophosphatidylinositol, an endogenous agonist for GPR55. Prostaglandins Other Lipid Mediat..

[9] Chiurchiù V., Leuti A., Maccarrone M. (2018). Bioactive Lipids and Chronic Inflammation: Managing the Fire Within. Front. Immunol..

[10] Falasca M., Ferro R. (2016). Role of the lysophosphatidylinositol/GPR55 axis in cancer. Adv. Biol. Regul..

[11] Alhouayek M., Masquelier J., Muccioli G.G. (2018). Lysophosphatidylinositols, from Cell Membrane Constituents to GPR55 Ligands. Trends Pharmacol. Sci..

[12] Kihara Y., Maceyka M., Spiegel S., Chun J. (2014). Lysophospholipid receptor nomenclature review: IUPHAR Review 8. Br. J. Pharmacol..

[13] Oka S., Nakajima K., Yamashita A., Kishimoto S., Sugiura T. (2007). Identification of GPR55 as a lysophosphatidylinositol receptor. Biochem. Biophys. Res. Commun..

[14] Chiurchiù V., Lanuti M., de Bardi M., Battistini L., Maccarrone M. (2015). The differential characterization of GPR55 receptor in human peripheral blood reveals a distinctive expression in monocytes and NK cells and a proinflammatory role in these innate cells. Int. Immunol..

[15] Hofmann N.A., Yang J., Trauger S.A., Nakayama H., Huang L., Strunk D., Moses M.A., Klagsbrun M., Bischoff J., Graier W.F. (2015). The GPR 55 agonist, L-α-lysophosphatidylinositol, mediates ovarian carcinoma cell-induced angiogenesis. Br. J. Pharmacol..

[16] Piñeiro R., Maffucci T., Falasca M. (2011). The putative cannabinoid receptor GPR55 defines a novel autocrine loop in cancer cell proliferation. Oncogene.

[17] Sutphen R., Xu Y., Wilbanks G.D., Fiorica J., Grendys E.C., LaPolla J.P., Arango H., Hoffman M.S., Martino M., Wakeley K. (2004). Lysophospholipids are potential biomarkers of ovarian cancer. Cancer Epidemiol. Biomarkers. Prev..

[18] Balenga N.A., Aflaki E., Kargl J., Platzer W., Schröder R., Blättermann S., Kostenis E., Brown A.J., Heinemann A., Waldhoer M. (2011). GPR55 regulates cannabinoid 2 receptor-mediated responses in human neutrophils. Cell Res..

[19] Kurano M., Kobayashi T., Sakai E., Tsukamoto K., Yatomi Y. (2021). Lysophosphatidylinositol, especially albumin-bound form, induces inflammatory cytokines in macrophages. FASEB J..

[20] Dráber P., Sulimenko V., Dráberová E. (2012). Cytoskeleton in mast cell signaling. Front. Immunol..

[21] Lazki-Hagenbach P., Klein O., Sagi-Eisenberg R. (2021). The actin cytoskeleton and mast cell function. Curr. Opin. Immunol..

[22] Cantarella G., Scollo M., Lempereur L., Saccani-Jotti G., Basile F., Bernardini R. (2011). Endocannabinoids inhibit release of nerve growth factor by inflammation-activated mast cells. Biochem. Pharmacol..

[23] Espinosa-Riquer Z.P., Ibarra-Sánchez A., Vibhushan S., Bratti M., Charles N., Blank U., Rodríguez-Manzo G., González-Espinosa C. (2019). TLR4 Receptor Induces 2-AG-Dependent Tolerance to Lipopolysaccharide and Trafficking of CB2 Receptor in Mast Cells. J. Immunol..

[24] Cruz S.L., Sánchez-Miranda E., Castillo-Arellano J.I., Cervantes-Villagrana R.D., Ibarra-Sánchez A., González-Espinosa C. (2018). Anandamide inhibits FcεRI-dependent degranulation and cytokine synthesis in mast cells through CB(2) and GPR55 receptor activation. Possible involvement of CB(2)-GPR55 heteromers. Int. Immunopharmacol..

[25] Jolly P.S., Bektas M., Olivera A., Gonzalez-Espinosa C., Proia R.L., Rivera J., Milstien S., Spiegel S. (2004). Transactivation of sphingosine-1-phosphate receptors by FcepsilonRI triggering is required for normal mast cell degranulation and chemotaxis. J. Exp. Med..

[26] Kulinski J.M., Muñoz-Cano R., Olivera A. (2016). Sphingosine-1-phosphate and other lipid mediators generated by mast cells as critical players in allergy and mast cell function. Eur. J. Pharmacol..

[27] Klein O., Krier-Burris R.A., Lazki-Hagenbach P., Gorzalczany Y., Mei Y., Ji P., Bochner B.S., Sagi-Eisenberg R. (2019). Mammalian diaphanous-related formin 1 (mDia1) coordinates mast cell migration and secretion through its actin-nucleating activity. J. Allergy Clin. Immunol..

[28] Zhou X.L., Guo X., Song Y.P., Zhu C.Y., Zou W. (2018). The LPI/GPR55 axis enhances human breast cancer cell migration via HBXIP and p-MLC signaling. Acta Pharmacol. Sin..

[29] Segura-Villalobos D., Ramírez-Moreno I.G., Martínez-Aguilar M., Ibarra-Sánchez A., Muñoz-Bello J.O., Anaya-Rubio I., Padilla A., Macías-Silva M., Lizano M., González-Espinosa C. (2022). Mast Cell-Tumor Interactions: Molecular Mechanisms of Recruitment, Intratumoral Communication and Potential Therapeutic Targets for Tumor Growth. Cells.

[30] Halova I., Draberova L., Draber P. (2012). Mast cell chemotaxis—Chemoattractants and signaling pathways. Front. Immunol..

[31] Poole T.J., Zetter B.R. (1983). Stimulation of rat peritoneal mast cell migration by tumor-derived peptides. Cancer Res..

[32] Zhang J., Childress S., Libchaber A., Shelley M. (2000). Flexible filaments in a flowing soap film as a model for one-dimensional flags in a two-dimensional wind. Nature.

[33] Huang B., Lei Z., Zhang G.M., Li D., Song C., Li B., Liu Y., Yuan Y., Unkeless J., Xiong H. (2008). SCF-mediated mast cell infiltration and activation exacerbate the inflammation and immunosuppression in tumor microenvironment. Blood.

[34] Zhu X.Q., Lv J.Q., Lin Y., Xiang M., Gao B.H., Shi Y.F. (2007). Expression of chemokines CCL5 and CCL11 by smooth muscle tumor cells of the uterus and its possible role in the recruitment of mast cells. Gynecol. Oncol..

[35] Kryczek I., Lange A., Mottram P., Alvarez X., Cheng P., Hogan M., Moons L., Wei S., Zou L., Machelon V. (2005). CXCL12 and vascular endothelial growth factor synergistically induce neoangiogenesis in human ovarian cancers. Cancer Res..

[36] Põlajeva J., Sjösten A.M., Lager N., Kastemar M., Waern I., Alafuzoff I., Smits A., Westermark B., Pejler G., Uhrbom L. (2011). Mast cell accumulation in glioblastoma with a potential role for stem cell factor and chemokine CXCL12. PLoS ONE.

[37] Weller C.L., Collington S.J., Hartnell A., Conroy D.M., Kaise T., Barker J.E., Wilson M.S., Taylor G.W., Jose P.J., Williams T.J. (2007). Chemotactic action of prostaglandin E2 on mouse mast cells acting via the PGE2 receptor 3. Proc. Natl. Acad. Sci. USA.

[38] Cabanillas-Saez A., Schalper J.A., Nicovani S.M., Rudolph M.I. (2002). Characterization of mast cells according to their content of tryptase and chymase in normal and neoplastic human uterine cervix. Int. J. Gynecol. Cancer.

[39] Oka S., Kimura S., Toshida T., Ota R., Yamashita A., Sugiura T. (2010). Lysophosphatidylinositol induces rapid phosphorylation of p38 mitogen-activated protein kinase and activating transcription factor 2 in HEK293 cells expressing GPR55 and IM-9 lymphoblastoid cells. J. Biochem..

[40] Henstridge C.M., Balenga N.A., Ford L.A., Ross R.A., Waldhoer M., Irving A.J. (2009). The GPR55 ligand L-alpha-lysophosphatidylinositol promotes RhoA-dependent Ca^2+^ signaling and NFAT activation. FASEB J..

[41] Fontemaggi G. (2023). Non-coding RNA regulatory networks in post-transcriptional regulation of VEGFA in cancer. IUBMB Life.

[42] Khera T.K., Dick A.D., Nicholson L.B. (2010). Mechanisms of TNFα regulation in uveitis: Focus on RNA-binding proteins. Prog. Retin. Eye Res..

[43] Hong H., Yoon B., Ghil S. (2021). Interactions between lysophosphatidylinositol receptor GPR55 and sphingosine-1-phosphate receptor S1P(5) in live cells. Biochem. Biophys. Res. Commun..

[44] Bang G., Ghil S. (2021). BRET analysis reveals interaction between the lysophosphatidic acid receptor LPA2 and the lysophosphatidylinositol receptor GPR55 in live cells. FEBS Lett..

[45] Balenga N.A., Martínez-Pinilla E., Kargl J., Schröder R., Peinhaupt M., Platzer W., Bálint Z., Zamarbide M., Dopeso-Reyes I.G., Ricobaraza A. (2014). Heteromerization of GPR55 and cannabinoid CB2 receptors modulates signalling. Br. J. Pharmacol..

[46] Moreno E., Andradas C., Medrano M., Caffarel M.M., Pérez-Gómez E., Blasco-Benito S., Gómez-Cañas M., Pazos M.R., Irving A.J., Lluís C. (2014). Targeting CB2-GPR55 receptor heteromers modulates cancer cell signaling. J. Biol. Chem..

[47] Wang Y., Matsushita K., Jackson J., Numata T., Zhang Y., Zhou G., Tsai M., Galli S.J. (2021). Transcriptome programming of IL-3-dependent bone marrow-derived cultured mast cells by stem cell factor (SCF). Allergy.

[48] Xu L., Yi H.G., Wu Z., Han W., Chen K., Zang M., Wang D., Zhao X., Wang H., Qu C. (2015). Activation of mucosal mast cells promotes inflammation-related colon cancer development through recruiting and modulating inflammatory CD11b(+)Gr1(+) cells. Cancer Lett..

[49] Groll T., Silva M., Sarker R.S.J., Tschurtschenthaler M., Schnalzger T., Mogler C., Denk D., Schölch S., Schraml B.U., Ruland J. (2022). Comparative Study of the Role of Interepithelial Mucosal Mast Cells in the Context of Intestinal Adenoma-Carcinoma Progression. Cancers.

[50] Meurer S.K., Neß M., Weiskirchen S., Kim P., Tag C.G., Kauffmann M., Huber M., Weiskirchen R. (2016). Isolation of Mature (Peritoneum-Derived) Mast Cells and Immature (Bone Marrow-Derived) Mast Cell Precursors from Mice. PLoS ONE.

[51] Madera-Salcedo I.K., Cruz S.L., Gonzalez-Espinosa C. (2013). Morphine prevents lipopolysaccharide-induced TNF secretion in mast cells blocking IκB kinase activation and SNAP-23 phosphorylation: Correlation with the formation of a β-arrestin/TRAF6 complex. J. Immunol..

[52] Tanaka S., Furuta K. (2021). Roles of IgE and Histamine in Mast Cell Maturation. Cells.

[53] Saitoh S., Arudchandran R., Manetz T.S., Zhang W., Sommers C.L., Love P.E., Rivera J., Samelson L.E. (2000). LAT is essential for Fc(epsilon)RI-mediated mast cell activation. Immunity.

[54] Jiménez-Andrade G.Y., Ibarra-Sánchez A., González D., Lamas M., González-Espinosa C. (2013). Immunoglobulin E induces VEGF production in mast cells and potentiates their pro-tumorigenic actions through a Fyn kinase-dependent mechanism. J. Hematol. Oncol..

[55] Hovey R.C., Goldhar A.S., Baffi J., Vonderhaar B.K. (2001). Transcriptional regulation of vascular endothelial growth factor expression in epithelial and stromal cells during mouse mammary gland development. Mol. Endocrinol..

[56] Dasgupta P., Betts V., Rastogi S., Joshi B., Morris M., Brennan B., Ordonez-Ercan D., Chellappan S. (2004). Direct binding of apoptosis signal-regulating kinase 1 to retinoblastoma protein: Novel links between apoptotic signaling and cell cycle machinery. J. Biol. Chem..

[57] Clua P., Tomokiyo M., Tonetti F.R., Islam M.A., Castillo V.G., Marcial G., Salva S., Alvarez S., Takahashi H., Kurata S. (2020). The Role of Alveolar Macrophages in the Improved Protection against Respiratory Syncytial Virus and Pneu-mococcal Superinfection Induced by the Peptidoglycan of Lactobacillus rhamnosus CRL1505. Cells.

[58] Bernard A., Cohen R., Khuth S.T., Vedrine B., Verlaeten O., Akaoka H., Giraudon P., Belin M.F. (1999). Alteration of the leptin network in late morbid obesity induced in mice by brain infection with canine distemper virus. J. Virol..

