# Supplementary material for: Lysophosphatidylinositol Promotes Chemotaxis and Cytokine Synthesis in Mast Cells with Differential Participation of GPR55 and CB2 Receptors
Source: Int J Mol Sci. 2023 Mar 28;24(7):6316. doi: 10.3390/ijms24076316 (PMC10094727; doi:10.3390/ijms24076316)
Supplement: Supplementary file 1 [file ijms-24-06316-s001.zip › SUPPL FIG 1.pdf]

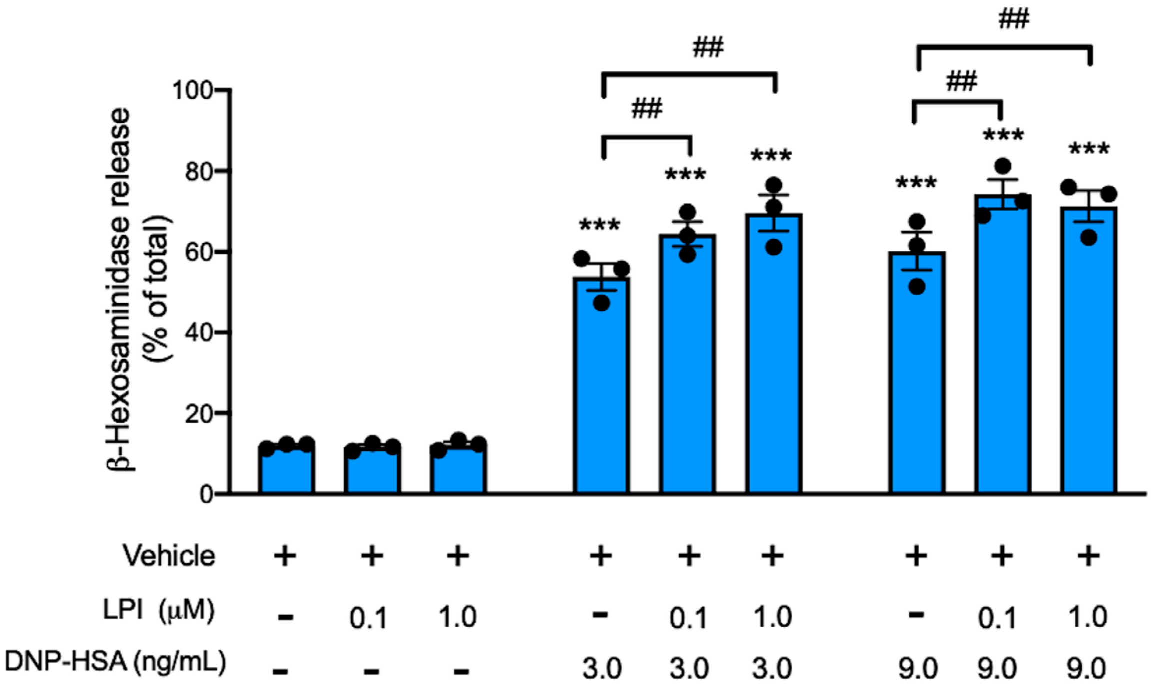

Figure S1. β-Hexosaminidase release in response to LPI alone or in the presence of two sub-optimal antigen concentrations in BMMCs. When required, LPI was added two minutes before DNP-HSA., *** $p < 0.001$ vs. vehicle-treated cells in the absence of antigen; ## $p < 0.01$ vs. vehicle pre-treated cells in the presence of antigen.
